# Enhancing drought stress tolerance and growth promotion in chiltepin pepper (*Capsicum annuum* var. *glabriusculum*) through native *Bacillus* spp.

**DOI:** 10.1038/s41598-024-65720-y

**Published:** 2024-07-04

**Authors:** Maribel Mendoza-Alatorre, Rocío Infante-Ramírez, María Olga González-Rangel, Guadalupe Virginia Nevárez-Moorillón, María del Carmen González-Horta, Jared Hernández-Huerta, Ma. Carmen E. Delgado-Gardea

**Affiliations:** 1 Facultad de Ciencias Químicas, Universidad Autónoma de Chihuahua, Circuito Nuevo Campus, Chihuahua, Chihuahua Mexico; 2 Facultad de Ciencias Agrotecnológicas, Universidad Autónoma de Chihuahua, Campus 1, Chihuahua, Chihuahua Mexico

**Keywords:** Environmental sciences, Biotechnology, Plant biotechnology

## Abstract

The drought can cause a decrease in food production and loss of biodiversity. In northern Mexico, an arid region, the chiltepin grows as a semi-domesticated crop that has been affected in its productivity and yield. An alternative to mitigate the effect of drought and aid in its conservation could be using Plant Growth-Promoting Bacteria (PGPB). The present study evaluated the capacity of native *Bacillus* spp., isolated from arid soils, as PGPBs and drought stress tolerance inducers in chiltepin under controlled conditions. Chiltepin seeds and seedlings were inoculated with native strains of *Bacillus* spp. isolated from arid soils, evaluating germination, vegetative, and drought stress tolerance parameters. The PGPBs improved vegetative parameters such as height, stem diameter, root length, and slenderness index in vitro*. B. cereus* (Bc25-7) improved in vitro survival of stressed seedlings by 68% at −1.02 MPa. Under greenhouse conditions, seedlings treated with PGPBs exhibited increases in root length (9.6%), stem diameter (13.68%), leaf fresh weight (69.87%), and chlorophyll content (38.15%). Bc25-7 alleviated severe water stress symptoms (7 days of water retention stress), and isolates *B. thuringiensis* (Bt24-4) and *B. cereus* (Bc25-7, and Bc30-2) increased Relative Water Content (RWC) by 51%. Additionally, the treated seeds showed improved germination parameters with a 46.42% increase in Germination Rate (GR). These findings suggest that using PGPBs could be an alternative to mitigate the effect of drought on chiltepin.

## Introduction

The drought is a problem that affects various regions of the world. It is one of the main limitations of modern agriculture, causing severe physiological stress in plants and compromising food production, as well as the loss of biodiversity^1–4^. In the last decade, global economic losses in the agricultural sector reached 37 billion dollars^5^. The northern region of Mexico is highly vulnerable to water deficits^6^. experiencing prolonged drought conditions in the extreme and exceptional categories from July 2020 to 2022^7^. Thus, drought threatens the productivity of chiltepin in Chihuahua, Mexico. *Capsicum annuum* var. *glabriusculum,* commonly known as chiltepin, is a semi-domesticated chili pepper of gastronomic and cultural significance in Mexico^8^. Chiltepin is valued for its high genetic variability, translating into broad adaptability to several environmental conditions. Therefore, chiltepin represents a valuable reservoir of primary genes to improve pepper crop cultivation through enhanced resistance to phytopathogens, pests, and unfavorable abiotic conditions^9^. Thus, it is essential to develop preservation strategies to protect the biodiversity of wild species^10^. In addition, chiltepin has a high economic value (up to 1,200.00 pesos per kilogram^11^. and recently its demand has increased.

A sustainable alternative to mitigate drought stress effects in agriculture is the crop treatment with Plant Growth-Promoting Bacteria. PGPBs act through many interconnected and synergistic mechanisms of action. Among these are phytohormones production, siderophore production, ammonium production, phosphate solubilization, osmoprotectant production, and gene expression regulation associated with antioxidant activity^12–15^. The selection of PGPB native to arid and semi-arid regions is relevant compared to the use of commercial strains, due to its potential to favor adaptability to drought and the reduction of alteration in the soil microbiota^16,17^. Furthermore, Stegeilmer et al.^18^ the current need to characterize PGPBs with solid and repeatable experiments to identify isolates that increase in food production in the context of growing human population. Species from the genera *Azotobacter, Azospirillum, Bradyrhizobium, Curtobacterium, Enterobacter, Gluconacetobacter, Micrococcus, Moraxella, Myroides, Paenibacillus, Pantoea, Providencia, Pseudomonas,* and *Bacillus* have been reported as PGPBs and inducers of drought stress relief^4,19,20^. The interest in the use of *Bacillus* spp. in agriculture has gained significant importance as various studies demonstrate their potential in promoting growth and providing protection to crops such as cereals, forage crops, legumes, and vegetables under stress conditions from biotic and abiotic agents^21–25^.

The use of *Bacillus* spp. has been reported in various chili species. Yasin et al.^26^ used PGPB to evaluate salt stress tolerance in *C. annuum* L. plants. They demonstrated physiological and biochemical processes regulation and increased plant biomass production. In a similar study, the potential of *B. amyloliquefaciens* to maintain growth and enhance responses to water stress, salt stress, and high heavy metal concentrations in *C. annuum* cv. *Geumsugangsan* plants was demonstrated^27^. There is also limited evidence regarding using PGPB to promote growth in *C. annuum* var. *glabriusculum* plants^28,29^. To our knowledge, there is no study where *Bacillus* spp. is used as a drought stress mitigator in chiltepin. Therefore, this study aimed to evaluate the capacity of native *Bacillus* spp., isolated from arid soils, to promote growth and induce drought tolerance in chiltepin. This study could serve as a basis for creating a technological package that allows for the improvement of reintroduction, preservation, and sustainable exploitation of chiltepin in drought and climate change.

## Results

### Morphological and biochemical identification of bacteria

The native bacterial isolates from the agricultural area exhibited characteristics of the *Bacillus* genus. All the bacteria formed ellipsoidal endospores. Isolates presented circular to irregular colonies ranging from 2 to 7 mm in diameter, with a beige to whitish color, creamy consistency, and serrated, entire, or lobed edges. Most bacteria exhibited a flat elevation, rough texture, and opaque transparency, characteristic of the *Bacillus cereus* group. However, isolate Bs22-3 showed a convex elevation, smooth texture, and mucoid and transparent consistency, typical of the *Bacillus subtilis* group.

### Molecular identification of bacteria

From the 16S rRNA fragment sequencing, assembled sequences with an average length of 1450 bp were obtained. According to sequence evaluation, 91.6% of the isolates belong to the *Bacillus cereus* group, and only one isolate corresponds to the *Bacillus subtilis* group (Table 1). Isolates Bc25-3 and Bc30-1 showed 100% similarity to *B. cereus* (MT332156.1 and MN746202.1, respectively). Similarly, Bc16-4 (MK780061.1), Bc16-5 (MK780061.1), Bc25-4 (KT720292.1), Bc25-7 (MT492023.1), and Bc30-2 (MK592620.1) were identified as *B. cereus* due to having over 99% similarity. Otherwise, isolates Bt22-1, Bt24-4 and Bt25-6 showed 99% similarity to *B. thuringiensis* (OM648228.1, MZ067912.1 and OP359440.1, respectively). Isolate Bw19-1 was identified as *B. wiedmannii* due to presenting 99% similarity with MT124531.1. Finally, isolate Bs22-3 revealed 100% similarity with *B. subtilis* (KU922339.1).

**Table 1:** Sequence analysis by 16S rRNA fragment and similarity with homologous sequences available in the NCBI GenBank.

Isolate	Identified as	Accession	% Similarity	Accession (Reference sequence)	Nucleotide similarity
Bc16-4	*B. cereus*	OR533503	99.86	MK780061.1	1446/1448
Bc16-5	*B. cereus*	OR533504	99.86	MK780061.1	1449/1451
Bw19-1	*B. wiedmannii*	OR533505	99.86	MT124531.1	1448/1450
Bt22-1	*B. thuringiensis*	OR533506	99.79	OM648228.1	1456/1459
Bs22-3	*B. subtilis*	OR533507	100.00	KU922339.1	1450/1450
Bt24-4	*B. thuringiensis*	OR533508	99.51	MZ067912.1	1435/1442
Bc25-3	*B. cereus*	OR533509	100.00	MT332156.1	1447/1447
Bc25-4	*B. cereus*	OR533510	99.72	KT720292.1	1446/1450
Bt25-6	*B. thuringiensis*	OR533511	99.93	OP359440.1	1448/1449
Bc25-7	*B. cereus*	OR533512	99.93	MT492023.1	1451/1452
Bc30-1	*B. cereus*	OR533513	100.00	MN746202.1	1449/1449
Bc30-2	*B. cereus*	OR533514	99.93	MK592620.1	1440/1441

### Multifarious plant growth promoting traits

All isolates demonstrated characteristics related to plant growth promotion, such as the ability to solubilize phosphorus and zinc and produce ammonium, siderophores, and exopolysaccharides (EPS) as mechanisms for growth promotion (Table 2). There were no differences in the phosphorus solubilization index compared to the commercial control in any treatments, with a mean of 3.08. Regarding the zinc test, only the Bs22-3 isolate did not exhibit solubilization capability. At the same time, the rest of the treatments showed the same solubilization activity as the control BsQ, with a mean index of 2.80. Siderophore production was observed in all isolates. Bt24-4, Bc25-7, and Bc30-1 bacteria exhibited the highest production index values (2.79), statistically equal to the commercial control, while the other isolates showed 6.30% lower production than the commercial control. In this study, all *Bacillus* isolates tested positive in the Nessler test; however, the behavior was significantly different in the evaluated treatments. Most of the isolates produced ammonium higher than the control, except Bs22-3. It is essential to highlight that the Bt25-6 isolate produced 8 times more ammonium than the commercial strain BsQ (805.31%). Finally, all isolates can produce exopolysaccharides, but Bs22-3 exhibited the same capacity as the commercial control. At the same time, the rest of the treatments showed a 77.23% lower production capacity—the values obtained in the in vitro tests allowed classifying most of the isolates as potential PGPB.

**Table 2:** PSI, ZnSI, siderophore, NH_4_, and exopolysaccharides production by *Bacillus* spp. The average of 4 repetitions represents the data. Different letters indicate significant statistical differences according to the Scott Knott test (*p* ≤ 0.05).

Treatment	PSI	ZnSI	Siderophore production Index	Ammonium production (μmol/mL)	Exopolysaccharides production
BsQ	3.01 ± 0.28 a	2.88 ± 0.07 a	2.59 ± 0.06 a	0.41 ± 0.03 e	2.36 ± 0.16 a
Bc16-4	3.25 ± 0.31 a	2.81 ± 0.03 a	2.42 ± 0.06 b	3.51 ± 0.16 b	0.38 ± 0.04 c
Bc16-5	3.03 ± 0.32 a	2.72 ± 0.04 a	2.41 ± 0.11 b	3.05 ± 0.18 c	0.20 ± 0.04 c
Bw19-1	3.16 ± 0.27 a	2.72 ± 0.06 a	2.39 ± 0.06 b	3.50 ± 0.24 b	0.74 ± 0.03 b
Bt22-1	3.04 ± 0.24 a	3.04 ± 0.10 a	2.47 ± 0.06 b	3.29 ± 0.26 b	0.50 ± 0.03 c
Bs22-3	3.19 ± 0.16 a	1.00 ± 0.00 b	2.53 ± 0.12 b	0.44 ± 0.08 e	2.53 ± 0.22 a
Bt24-4	3.01 ± 0.26 a	2.84 ± 0.10 a	2.93 ± 0.07 a	1.60 ± 0.08 d	0.42 ± 0.08 c
Bc25-3	2.93 ± 0.17 a	3.07 ± 0.17 a	2.48 ± 0.07 b	3.58 ± 0.12 b	0.50 ± 0.07 c
Bc25-4	3.12 ± 0.43 a	2.59 ± 0.15 a	2.28 ± 0.04 b	2.99 ± 0.11 c	0.61 ± 0.05 b
Bt25-6	3.01 ± 0.24 a	2.68 ± 0.09 a	2.44 ± 0.08 b	5.32 ± 0.42 a	0.47 ± 0.06 c
Bc25-7	3.40 ± 0.27 a	2.79 ± 0.10 a	2.72 ± 0.07 a	3.29 ± 0.11 b	0.69 ± 0.03 b
Bc30-1	3.03 ± 0.33 a	2.64 ± 0.11 a	2.92 ± 0.10 a	3.33 ± 0.20 b	0.58 ± 0.03 b
Bc30-2	2.94 ± 0.52 a	2.93 ± 0.15 a	2.42 ± 0.05 b	2.86 ± 0.15 c	0.82 ± 0.02 b

### Germination promotion in chiltepin

Most *Bacillus* spp. isolates showed PGPB characteristics promoting germination and enhancing the vegetative parameters of chiltepin seedlings in vitro. *B. cereus* treatments (Bc16-5, Bc25-4, Bc25-7, and Bc30-2), as *B. subtilis* Bs22-3, and *B. thuringiensis*Bt24-4 treatments, increased the germination rate (GR) by 46.42% compared to untreated seeds (Table 3). Similarly, *B. cereus* isolates (Bc16-5, Bc25-4, Bc25-7, and Bc30-2), *B. thuringiensis* (Bt22-1, y Bt24-4), and *B. subtilis* Bs22-3 induced a 22.56% and 22.19% increase in the mean speed of germination (MSG) and germination speed index (GSI), respectively. In addition, seeds treated with Bs22-3, Bc25-4, Bc25-7, and Bc30-2 increased the vigor index (VI) by 65.16%, while treatments Bc16-5, Bs22-3, Bt24-4, Bc25-4, Bc25-7, and Bc30-2 increased the germination index (GI) by 100.14%. Furthermore, treatments Bc16-5, Bt22-1, Bs22-3, and Bc30-2 reduced the mean germination time (MGT) by 5.63% compared to the control.

**Table 3:** Germination parameters (GR, MSG, MGT, and GSI) and vigor, germination, and slenderness indices in *C. annuum* var. *glabriusculum* seeds treated with *Bacillus* spp. The average of 4 repetitions represents the data. Different letters indicate significant statistical differences according to the Scott Knott test (*p* ≤ 0.05).

Treatment	GR (%)	MSG (seeds/day)	MGT (days)	GSI	Vigor index	Germination index
C	56 ± 8.64 b	0.88 ± 0.14 b	7.45 ± 0.20 a	8.87 ± 1.40 b	647.11 ± 102.8 b	98.94 ± 7.28 b
BsQ	56 ± 14.61 b	0.85 ± 0.12 b	7.50 ± 0.36 a	8.50 ± 1.29 b	779.57 ± 162.1 b	105.59 ± 28.15 b
Bc16-4	64 ± 9.80 b	0.97 ± 0.17 b	7.45 ± 0.32 a	9.74 ± 1.69 b	807.60 ± 239.9 b	132.68 ± 31.17 b
Bc16-5	70 ± 11.55 a	1.15 ± 0.10 a	6.99 ± 0.14 b	11.54 ± 1.06 a	877.18 ± 173.2 b	217.62 ± 40.51 a
Bw19-1	60 ± 11.31 b	0.87 ± 0.11 b	7.34 ± 0.17 a	8.70 ± 1.14 b	579.87 ± 109.4 b	62.64 ± 14.42 b
Bt22-1	62 ± 11.37 b	1.05 ± 0.21 a	7.02 ± 0.36 b	10.58 ± 2.15 a	818.24 ± 209.2 b	149.17 ± 93.96 b
Bs22-3	68 ± 7.30 a	1.02 ± 0.10 a	7.18 ± 0.28 b	10.25 ± 1.03 a	1068.36 ± 138.6 a	236.34 ± 22.83 a
Bt24-4	71 ± 3.83 a	1.00 ± 0.13 a	7.36 ± 0.13 a	10.05 ± 1.32 a	864.53 ± 117.9 b	186.63 ± 20.80 a
Bc25-3	59 ± 8.25 b	0.89 ± 0.07 b	7.46 ± 0.10 a	8.95 ± 0.72 b	732.24 ± 109.1 b	110.89 ± 15.84 b
Bc25-4	74 ± 2.31 a	1.08 ± 0.00 a	7.26 ± 0.08 a	10.80 ± 0.08 a	961.44 ± 64.0 a	199.48 ± 32.26 a
Bt25-6	52 ± 11.78 b	0.85 ± 0.12 b	7.33 ± 0.19 a	8.54 ± 1.25 b	678.09 ± 112.1 b	92.08 ± 21.23 b
Bc25-7	74 ± 6.93 a	1.09 ± 0.10 a	7.27 ± 0.16 a	10.99 ± 0.99 a	1100.06 ± 189.8 a	180.54 ± 59.67 a
Bc30-1	60 ± 8.64 b	0.89 ± 0.13 b	7.33 ± 0.08 a	8.93 ± 1.34 b	884.42 ± 267.0 b	133.78 ± 36.55 b
Bc30-2	73 ± 9.98 a	1.16 ± 0.14 a	7.02 ± 0.10 b	11.66 ± 1.47 a	1145.44 ± 157.5 a	167.51 ± 35.77 a

Regarding vegetative parameters, treatments Bs22-3, Bc25-7, Bc30-1, Bc30-2, and the control commercial isolate BsQ increased the height by 27.96% (Table 4). Similarly, treatments Bc16-5, Bt22-1, Bs22-3, Bt24-4, Bc25-4, Bc25-7, Bc30-1, and Bc30-2 increased root length by 44.32%. Moreover, total fresh weight (FW) increased by 20% in all treatments except for Bw19-1 and Bt25-6. Additionally, there were increases of 12.13% in stem diameter in seedlings treated with Bc16-4, Bt22-1, Bs22-3, Bt24-4, Bc25-4, and Bc30-1 compared to uninoculated plants (C). Similarly, treatments Bc16-4, Bw19-1, and Bt24-4 reduced the slenderness index by 11.77%.

**Table 4:** Vegetative parameters (height, root length, stem diameter, FW, and slenderness index) of *C. anuum* var. *glabriusculum* seedlings treated with *Bacillus* spp. on 19 DAS. The average of 4 repetitions represents the data. Different letters indicate significant statistical differences according to the Scott Knott test (*p* ≤ 0.05).

Treatment	Height (mm)		Root length (mm)		Stem diameter (mm)		FW (mg)		Slenderness index	
C	11.70 ± 2.80	b	9.74 ± 3.26	d	0.81 ± 0.251	b	17.16 ± 3.89	b	1.50 ± 0.44	b
BsQ	14.13 ± 2.52	a	10.26 ± 3.10	d	0.81 ± 0.154	b	20.92 ± 4.62	a	1.76 ± 0.35	a
Bc16-4	12.51 ± 3.17	b	11.27 ± 3.12	d	0.91 ± 0.185	a	19.69 ± 4.41	a	1.39 ± 0.38	c
Bc16-5	12.59 ± 2.88	b	15.49 ± 4.16	b	0.82 ± 0.089	b	20.11 ± 4.25	a	1.53 ± 0.31	b
Bw19-1	9.70 ± 1.69	c	5.82 ± 2.32	e	0.83 ± 0.161	b	16.82 ± 3.45	b	1.20 ± 0.28	c
Bt22-1	13.28 ± 2.77	b	12.36 ± 5.38	c	0.89 ± 0.131	a	20.54 ± 4.09	a	1.51 ± 0.39	b
Bs22-3	15.72 ± 2.15	a	18.10 ± 5.36	a	0.91 ± 0.078	a	21.80 ± 3.68	a	1.74 ± 0.32	a
Bt24-4	12.12 ± 1.60	b	13.95 ± 2.88	c	0.88 ± 0.084	a	21.15 ± 4.83	a	1.38 ± 0.23	c
Bc25-3	15.56 ± 2.73	b	10.36 ± 3.56	d	0.81 ± 0.109	b	19.30 ± 4.61	a	1.55 ± 0.35	b
Bc25-4	12.98 ± 2.63	b	14.70 ± 4.74	b	0.92 ± 0.119	a	21.54 ± 4.20	a	1.50 ± 0.37	b
Bt25-6	13.35 ± 3.63	b	9.72 ± 3.57	d	0.85 ± 0.159	b	18.09 ± 3.63	b	1.60 ± 0.52	b
Bc25-7	14.80 ± 2.76	a	13.11 ± 4.38	c	0.82 ± 0.109	b	21.48 ± 3.57	a	1.80 ± 0.36	a
Bc30-1	14.57 ± 2.86	a	12.08 ± 2.92	c	0.94 ± 0.193	a	20.04 ± 3.27	a	1.60 ± 0.48	b
Bc30-2	15.64 ± 1.83	a	12.67 ± 3.61	c	0.79 ± 0.113	b	21.74 ± 3.89	a	2.01 ± 0.41	a

**Figure 1:**
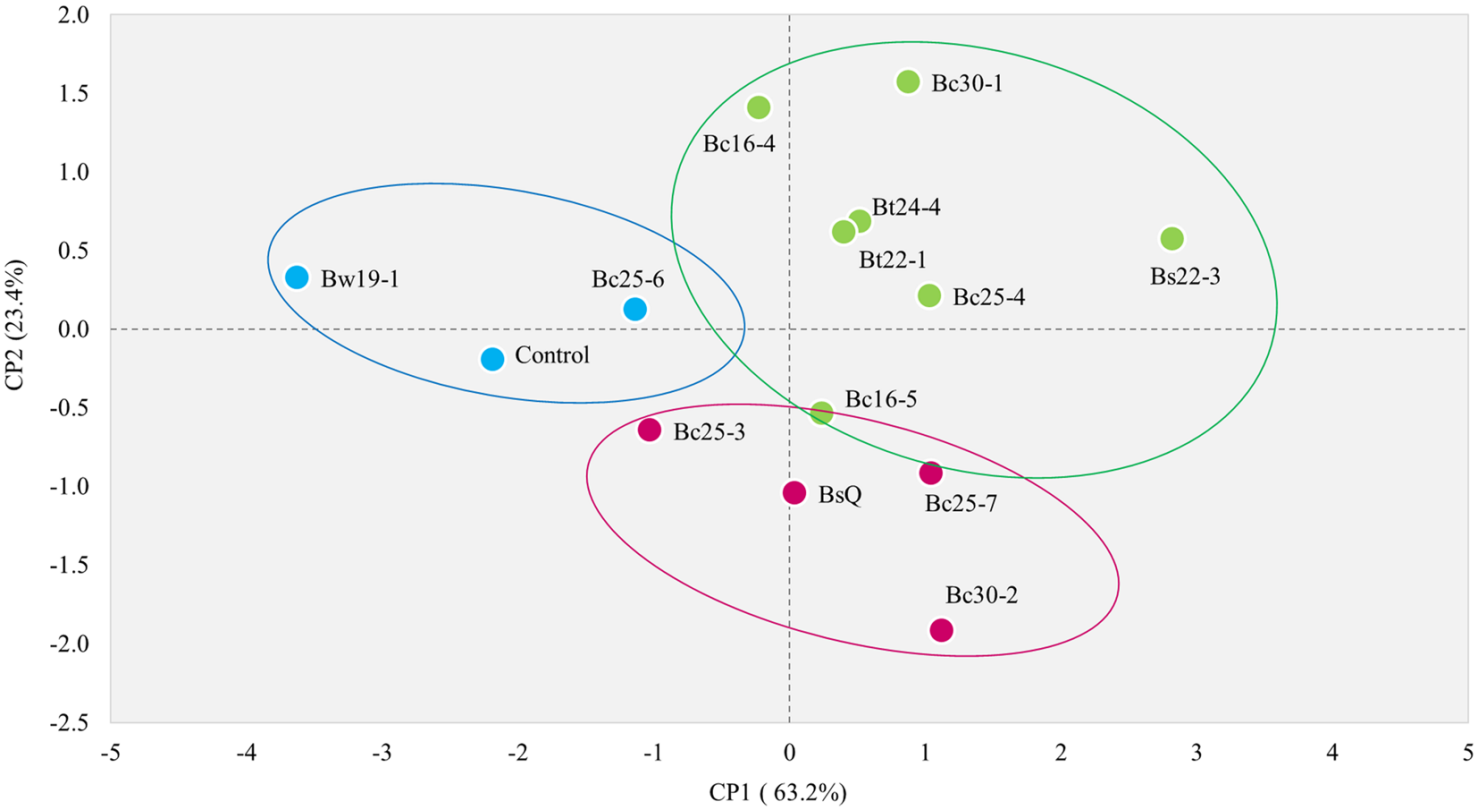
Principal component analysis (PCA) and k-means cluster analysis of vegetative parameters (height, total fresh weight, root length, stem diameter) in *C. annuum* var. *glabriusculum* seedlings treated with *Bacillus* spp. as Plant Growth-Promoting Bacteria in vitro. Control, Bc16-4-Bc30-2 = *B. cereus* isolates, Bt22-1-Bt25-6 = *B. thuringiensis* isolates, Bs22-3 = *B. subtilis* isolate, Bw19-1 = *B. wiedmannii* isolate.

The PCA analysis of vegetative parameters from the in vitro germination promotion trial, conducted to determine the best performance as PGPB, explains 86.6% of the variability in the data. PC1 explained 63.2% of the variation, and PC2 explained 23.4% of the variation in the data (Fig. 1). According to the k-means cluster analysis, the group with the most positive effect on growth consisted of the bacteria Bs22-3, Bc30-1, Bt24-4, Bt22-1, Bc16-4, and Bc16-5, corresponding to the parameters of FW, height, root length, and stem diameter. Otherwise, bacteria Bc25-3, Bc25-7, Bc30-2, and the commercial control, showed moderate performance in promoting growth across all parameters. Finally, bacteria Bt25-6, Bw19-1, and untreated seedlings formed the group with the least potential as PGPR in chiltepin.

### *Drought tolerance under *in vitro* conditions*

#### Drought tolerance potential of *Bacillus* isolates

Before being used as inducers of tolerance, the strains resistance to water stress was evaluated under controlled conditions using the PEG test. A minimum survival rate of 60% relative to the control (P0) was considered to classify tolerant bacteria. Thirty percent of the isolates were sensitive to P30, while 38% exhibited high tolerance (Table 5). Treatments Bt25-6 and Bc30-2 were entirely tolerant for the highest evaluated concentration (P50). All isolates remained viable until exposure to P40, except for Bs22-3 and the control strain BsQ.

**Table 5:** *Bacillus* spp. isolates tolerant to concentrations 0–50% polyethylene glycol (PEG) at 96 h of exposure in vitro. The average of 3 replicates represents data. P0 = Control, P10-P50 = PEG concentrations (10–50%).

PEG concentration	Tolerant isolates	Tolerant isolates number
P10	BsQ, Bc16-5, Bt22-1, Bs22-3, Bt24-4, Bc25-3, Bc25-4, Bc25-7	8
P20	BsQ, Bc16-5, Bt22-1, Bt24-4, Bc25-3, Bc25-4, Bt25-6	7
P30	BsQ, Bc16-5, Bt22-1, Bt24-4, Bc25-4	5
P40	BsQ, Bc25-3	2
P50	Bt25-6, Bc30-2	2

#### In vitro* evaluation of stress tolerance induction in chiltepin seedlings*

Once the bacterial tolerance to stress was tested, stress tolerance induction was evaluated in chiltepin seedlings. All treatments improved the survival of seedlings stressed with P30 (Table 6). Treatments Bc25-3 and Bc25-7 increased the survival rate to 80% and 90%, respectively, compared to the negative control (PEG 30%). Similarly, treatments Bt25-6 and Bc30-2 induced a survival rate of 70%. In addition, the BsQ treatment had a survival rate of 40%. Treatments Bc25-3, Bc25-7, Bc30-2, and Bt25-6 exhibited normal morphological characteristics. At the same time, the rest of the stressed seedlings showed leaf chlorosis, stem deformation, necrotic areas in the radicle, and loss of turgidity (Supplementary Fig. S1). Regarding vegetative parameters, seedlings treated with bacteria showed significant differences compared to the negative control. Treatments Bw19-1 and Bt25-6 had a 27.21% higher height than the negative control. Otherwise, Bt24-4, Bt25-6, Bc25-3, Bc25-4, Bc25-7, and Bc30-2 increased stem diameter by 21.92%. Regarding the slenderness index, there was a 30.28% increase with the Bw19-1 isolate and a 19.29% decrease in treatments Bt24-4, Bc25-3, Bc25-4, Bc25-7, and Bc30-2. Additionally, treatments, Bw19-1, Bt24-4, Bc16-5, Bc25-3, and Bc25-7 showed a 27.94% reduction in root length.

**Table 6:** Survival rate and vegetative parameters (height, root length, stem diameter, and slenderness index) of *C. annuum* var. *glabriusculum* seedlings under water stress (PEG 30%). The average of 9 repetitions represents data. Different letters indicate significant statistical differences according to the Scott Knott test (*p* ≤ 0.05).

Treatment	Survival rate (%)	Height (mm)		Root length (mm)		Stem diameter (mm)		Slenderness index	
C abs	100	13.13 ± 0.96	b	22.22 ± 4.03	a	0.77 ± 0.14	b	1.73 ± 0.34	a
C-	20	10.82 ± 2.42	c	19.35 ± 3.38	a	0.76 ± 0.06	b	1.42 ± 0.31	b
BsQ	40	13.37 ± 2.63	b	11.86 ± 1.03	c	0.75 ± 0.16	b	1.82 ± 0.49	a
Bc16-4	60	11.66 ± 1.75	c	20.50 ± 2.51	a	0.82 ± 0.08	b	1.43 ± 0.25	b
Bc16-5	40	11.72 ± 1.09	c	12.01 ± 2.89	c	0.81 ± 0.07	b	1.45 ± 0.16	b
Bw19-1	30	14.96 ± 1.89	a	9.94 ± 0.83	c	0.81 ± 0.14	b	1.85 ± 0.24	a
Bt22-1	30	11.61 ± 1.88	c	23.39 ± 6.39	a	0.80 ± 0.09	b	1.44 ± 0.23	b
Bs22-3	40	11.12 ± 1.34	c	18.44 ± 6.02	a	0.80 ± 0.07	b	1.38 ± 0.18	b
Bt24-4	50	9.90 ± 0.84	c	15.62 ± 3.87	b	0.93 ± 0.10	a	1.07 ± 0.15	c
Bc25-3	80	10.47 ± 2.06	c	15.76 ± 5.47	b	0.90 ± 0.08	a	1.17 ± 0.26	c
Bc25-4	40	10.60 ± 1.74	c	20.35 ± 2.90	a	0.95 ± 0.10	a	1.12 ± 0.19	c
Bt25-6	70	12.57 ± 1.71	b	18.47 ± 3.55	a	0.89 ± 0.11	a	1.42 ± 0.30	b
Bc25-7	90	10.94 ± 1.04	c	16.38 ± 3.04	b	0.92 ± 0.09	a	1.19 ± 0.14	c
Bc30-1	40	10.34 ± 0.88	c	22.38 ± 4.25	a	0.77 ± 0.09	b	1.35 ± 0.14	b
Bc30-2	70	11.48 ± 1.58	c	19.23 ± 3.90	a	0.97 ± 0.07	a	1.18 ± 0.20	c

### Growth promotion and drought tolerance in greenhouse conditions

#### Growth promotion

**Figure 2:**
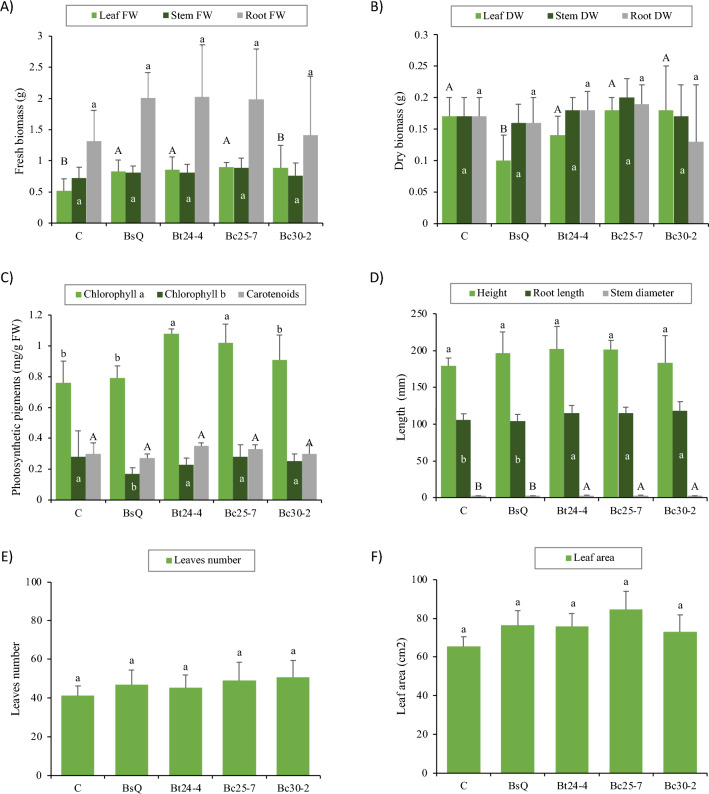
Vegetative parameters (fresh and dry biomass, photosynthetic pigments, height, root length, stem diameter, leaves number and leaf area) of chiltepin seedlings treated with *Bacillus* spp. on day 42 days after transplant. The average of 6 repetitions represents the data. Different letters indicate significant statistical differences according to the Scott Knott test (*p* ≤ 0.05).

After the in vitro evaluation of germination tests and stress tolerance, two strains of *B. cereus* (Bc25-7 and Bc30-2) and one strain of *B. thuringiensis* (Bt24-4) were selected to determine their effectiveness under greenhouse conditions. All isolates induced a significant increase in at least one of the vegetative parameters (Fig. 2, Supplementary Fig. S2). Treatments Bt24-4, Bc25-7, and Bc30-2 increased root length by 9.6% compared to the control (uninoculated seedlings) and 11.5% compared to the commercial isolate. Stem diameter increased by 13.68% with treatments Bt24-4 and Bc25-7 compared to the control and 6.02% compared to the commercial isolate. Likewise, leaf fresh weight increased by 69.87% with isolates Bt24-4, Bc25-7, and Bc30-2 compared to the control. Besides, chlorophyll a content increased by 38.15% in treatments Bt24-4 and Bc25-7 compared to the control and 32.9% compared to the commercial strain. In contrast, parameters such as height, number of leaves, leaf area, stem and root fresh weight, dry weight, chlorophyll b, and carotenoids did not show significant differences compared to the control.

**Figure 3:**
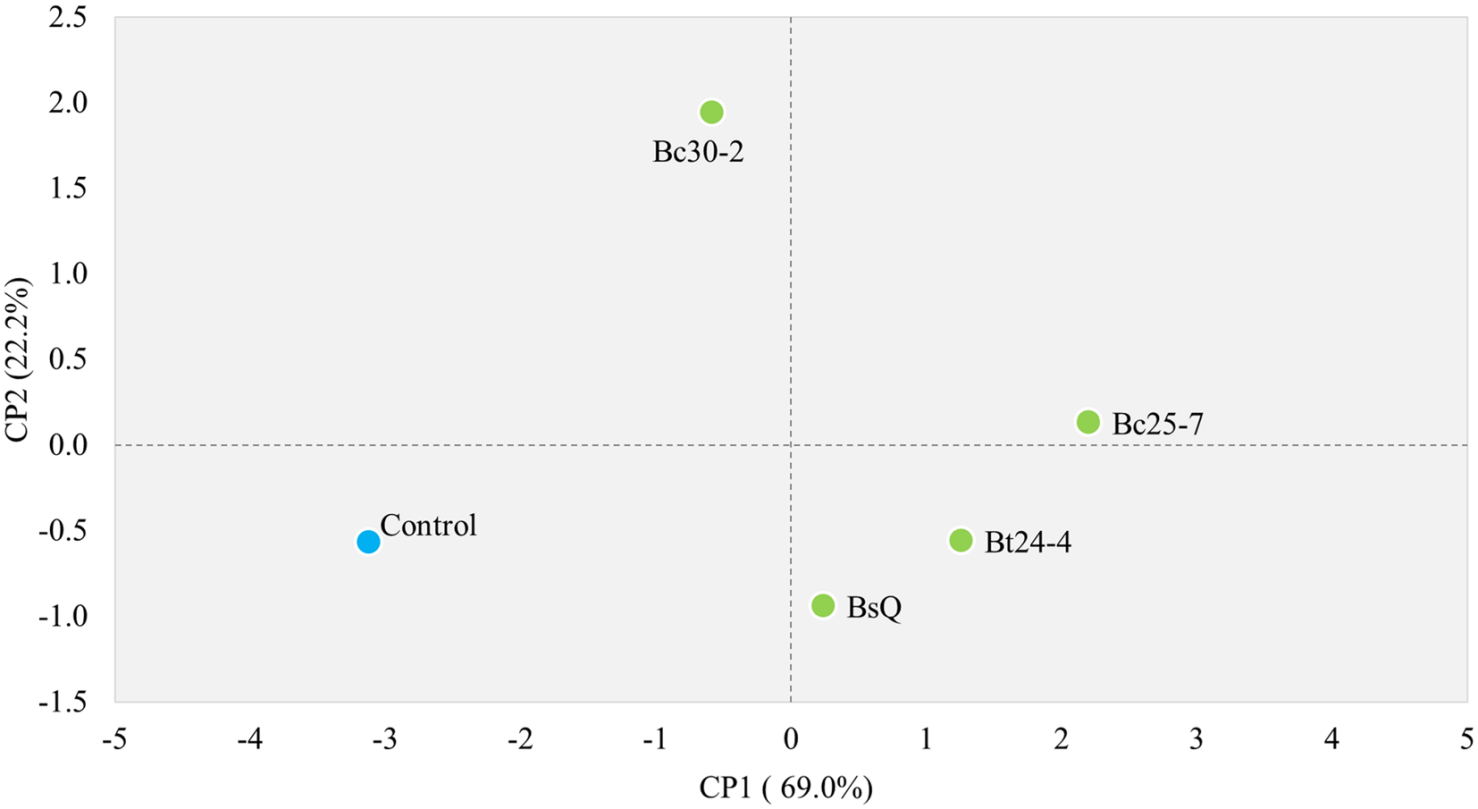
Principal component analysis (PCA) of vegetative parameters (height, total fresh weight, root length, stem diameter, leaf area, and leaves number) in *C. annuum* var. *glabriusculum* seedlings treated with *Bacillus* spp. as Plant Growth-Promoting Bacteria developed in greenhouse conditions. Control, BsQ = *B. subtilis* QST 713 ®, Bc25-7-Bc30-2 = *B. cereus* isolates, Bt24-4 = *B. thuringiensis* isolate.

The PCA analysis of vegetative parameters from the greenhouse germination promotion trial, conducted to determine the best performance as PGPB in the greenhouse, explains 91.2% of the variability in the data. PC1 explained 69.0% of the variation, and PC2 explained 22.2% of the variation in the data (Fig. 3). Treatment Bc25-7 exhibited the most positive effect on chiltepin growth across all evaluated vegetative parameters. Otherwise, the isolate Bt24-4 and the commercial control positively affected leaf area, fresh weight, stem diameter, and seedling height, while Bc30-2 mainly increased root length and the number of leaves.

### Drought tolerance in greenhouse conditions

The seedlings treated with Bc25-7 presented an alleviation in visual symptoms of wilting caused by severe water stress (loss of leaf turgidity and reduced growth rate) compared to non-inoculated seedlings (negative control), even though the substrate had similar moisture levels (Supplementary Fig. S3). Water stress led to a significant reduction in the relative water content (RWC) in chiltepin leaves. However, all isolates increased RWC by 51.28% compared to the negative control, exhibiting similar behavior to the commercial control (Table 7). Otherwise, applying water stress induced a significant increase in proline content (30 times higher) compared to the unstressed seedlings. However, isolates Bt24-4, Bc25-7, and Bc30-2 showed 50.36% less proline content in leaves compared to the negative control.

**Table 7:** RWC and proline in *C. annuum* var. *glabrisculum* leaves subjected to drought stress. Data are represented by the average of 6 and 3 repetitions, respectively. Different letters indicate significant statistical differences according to the Scott Knott test (*p* ≤ 0.05).

Treatment	Proline (µM / g of leaf)	RWC (%)
C abs	4.44 ± 0.70 c	100.04 ± 13.30 a
C Xp Amino®	136.60 ± 32.7 a	46.76 ± 10.18 b
C -	135.21 ± 1.09 a	28.56 ± 09.43 c
Bt24-4	63.03 ± 14.6 b	50.49 ± 12.45 b
Bc25-7	84.77 ± 14.1 b	57.71 ± 21.54 b
Bc30-2	53.55 ± 13.3 b	45.64 ± 07.58 b

## Discussion

The morphological and biochemical characterization of native bacterial isolates provides fundamental information about their phenotypic traits, aiding in the initial identification process. Observed characteristics such as colony morphology, color, texture, and spore formation indicated the *Bacillus* genus^30^. Molecular identification of bacteria by 16S rRNA offers a more precise and discriminatory approach, allowing differentiation of closely related species within the *Bacillus* genus^31^. Among the isolates identified as members of the *B. cereus* group, a high sequence similarity with reference strains further supports their classification. Specifically, isolates Bc25-3 and Bc30-1 exhibited complete similarity to *B. cereus* reference sequences, indicating a close phylogenetic relationship. In contrast, isolate Bs22-3 showed 100% similarity with *B. subtilis*, diverging from the predominant group of *B. cereus*. Furthermore, identifying isolate Bw19-1 as *B. wiedmannii* was less studied than other *Bacillus* species. *B. wiedmannii* isolation represents a notable contribution to understanding the microbial diversity of arid soil areas.

Improving crop germination processes remains critical for increasing productivity and addressing agronomic limitations^32^. Chiltepin, a wild chili species, poses challenges due to its low germination rates and high variability^9,33^. Therefore, the application of *Bacillus* spp. rhizobacteria in this study demonstrated significant improvements in chiltepin seedling germination parameters, offering a promising solution to these challenges. The findings of this study are consistent with previous research demonstrating the germination-promoting effects of *Bacillu*s spp. in various species within the *Capsicum* genus. For instance, Bolaños et al.^34^ observed a notable increase in germination rates in pepper seeds (*C. annuum* L.) after inoculation with *B. licheniformis* M2-7, while *B. subtilis* was found to enhance germination in "Kandil Dolma" peppers^35^. These observed improvements can be attributed to the production of phytohormones such as gibberellins, cytokinins, and auxins by *Bacillus* spp.^33^. These growth regulators play fundamental roles in seed germination by stimulating nutrient mobilization, hydrolyase synthesis, and early root development through cell division and enzymatic actions^36,37^. The significant reduction in mean germination time indicates accelerated germination, which is crucial, especially under stress conditions, as it increases the likelihood of successful seedling establishment and growth^32^. The observed reduction in MGT aligns with previous research, as reported by Sosa et al.^38^ and Dutta et al.^39^, who documented similar reductions in *C. chinense* Jacq. and *C. frutescens* L. seeds inoculated with *Bacillus* spp.

Furthermore, vigor and germination indices are essential indicators of seed potency and potential yield during germination^32,40^. The results found in this study were consistent with studies reported by Jayapala et al.^41^, who observed an increase in vigor and germination indices in chili plants through biopriming with *Bacillus* sp. BSp. Finally, the slenderness index, which reflects the quality and resilience of seedlings, revealed robust seedlings in a significant proportion of treatments after applying *Bacillus* spp.^42^. These findings suggest that *Bacillus* spp. isolates accelerate germination and facilitate robust seedling establishment by promoting root development and rapid shoot growth.

The positive effects observed in the growth of chiltepin plants in the greenhouse trial align with previous studies demonstrating the beneficial impact of *Bacillus* spp. on various plant species. The treated seedlings exhibited increased root length, stem diameter, leaf fresh weight, and chlorophyll content, indicating improved vegetative development and potential yield enhancement. This corroborates the findings of García et al.^29^, Kazerooni et al.^27^, and others who reported significant increases in root length and fresh weight in chili and other plant species after inoculation with *Bacillus* strains^27,29,43,44^. Such morphological improvements are crucial for enhancing nutrient uptake and overall plant vitality, impacting crop productivity^45^. Furthermore, increased chlorophyll content observed in treated chiltepin plants suggests enhanced photosynthetic activity, which is crucial for meeting elevated metabolic demands during critical stages such as flowering and fruiting^46,47^.

The underlying mechanisms behind these growth-promoting effects likely involve multiple pathways facilitated by rhizobacteria. Notably, the capacity to solubilize essential nutrients such as phosphorus and zinc from their non-assimilable forms into accessible forms represents a fundamental aspect of plant-microorganism interactions^48,49^. The solubilization of phosphorus and zinc by *Bacillus* spp. can be attributed to various mechanisms, including medium acidification, secretion of organic acids, and enzymatic activities, all of which contribute to improving nutrient availability in the rhizosphere^50,51^. Additionally, the production of ammonia by rhizobacteria serves as a vital nitrogen source for plants, stimulating root development and overall biomass accumulation^52,53^. The positive impact of ammonia production on chili plant growth observed in this study corroborates previous findings by Bhattacharyya et al*.*^52^, and Abdelwahed et al*.*^54^. regarding the role of *Bacillus* spp. in nitrogen supply and plant development. Furthermore, the production of siderophores by *Bacillus* strains represents another mechanism contributing to plant growth promotion. Siderophores enhance plant iron availability, aiding nutrient uptake and serving as a competitive advantage against phytopathogens^27,55^. The isolation of native *Bacillus* strains producing siderophores in this study suggests their potential as biocontrol agents and growth promoters in agricultural systems. Additionally, the production of exopolysaccharides by *Bacillus* spp. has significant implications for soil health and plant-microorganism interactions^56–59^. EPS plays various roles, including soil aggregation, water retention, and nutrient enrichment, ultimately facilitating root colonization and environmental stress mitigation in plants^13,60^. The growth-promoting traits the native *Bacillus* isolates tested underscore their potential as biofertilizers in chiltepin chili production systems. By enhancing nutrient availability and improving soil properties while mitigating environmental stress, these types of rhizobacteria offer sustainable alternatives to conventional fertilizers and pesticides, thus contributing to ecological balance and horticultural crop productivity^53,61^.

The rhizobacteria isolates displayed high tolerance up to an osmotic pressure of −1.75 MPa. Previous studies have reported varying degrees of drought tolerance in *Bacillus* spp. under similar conditions^62^For instance, Ashry et al.^63^ reported that 25% of isolates isolated from arid soils showed resistance to drought under different water potentials ranging from −0.15 to −1.2 MPa. In another study, *B. megaterium* and *B. licheniformis* were able to grow at the lowest water potential tested (−0.73 MPa^13^. This adaptation to water deficit is attributed to the genus ability of *Bacillus* to form endospores^63^. Other authors associate the resistance of *Bacillus* spp. and the amelioration of drought stress in plants with mechanisms such as EPS production, phytohormones, enzymes, and osmolytes^13,62^.^.^

The findings of this study provide insights into the underlying mechanisms of drought tolerance potential of *Bacillus* isolates and their effects on chiltepin seedlings under stress conditions. The observed results demonstrate the ability of *Bacillus* isolates to enhance drought tolerance in chiltepin seedlings, as evidenced in both in vitro and greenhouse experiments. The in vitro evaluation of stress induction in chiltepin seedlings revealed that specific *Bacillus* treatments significantly improved seedling survival rates under stress conditions. Treatments such as Bc25-3 and Bc25-7 showed notable increases in survival rates compared to the negative control (non-inoculated plants). This improvement in survival can be attributed to the ability of *Bacillus* isolates to induce stress tolerance mechanisms in plants. Similar findings have been reported in previous studies, where *Bacillus* treatments increased survival rates of various plant species under drought conditions^64–66^. Furthermore, the morphological characteristics of stressed seedlings treated with *Bacillus* isolates improved significantly compared to the negative control. Treatments such as Bc24-4, Bc25-3, Bc25-4, Bt25-6, Bc25-7, and Bc30-2 showed enhancements in height, stem diameter, and slenderness index, indicating a positive impact on seedling growth and development. These findings are consistent with previous research demonstrating the beneficial effects of *Bacillus* treatments on plant morphology under drought stress^67,68^.

Additionally, the greenhouse trial confirmed the effectiveness of *Bacillus* treatments in mitigating drought stress in chiltepin seedlings. The Bc25-7 treatment alleviated visual wilting symptoms and significantly increased the relative water content (RWC) in chiltepin leaves compared to uninoculated plants. According to categories described by Bandurska^69^, unstressed plants have an RWC in leaves above 90%, while plants under mild drought stress show values between 60 and 70%. An RWC between 40 and 60% indicates moderate water stress, and values below 40% correspond to severe stress. Thus, the treatment with *Bacillus* spp. they reduced the stress level in chiltepin seedlings from severe to moderate. These findings suggest that the tested native *Bacillus* isolates enhance drought tolerance in chiltepin seedlings by modulating physiological responses. Similar results have been reported in previous studies, where *Bacillus* treatments led to an increase in RWC and an increment in proline accumulation in plants subjected to drought stress^70,71^ The underlying mechanisms behind the observed effects of *Bacillus* treatments on drought tolerance in chiltepin seedlings are likely multifaceted. Previous research has suggested several potential mechanisms, including the production of bacterial biofilms, modulation of stomatal behavior, and activation of adaptive responses mediated by phytohormones such as abscisic acid (ABA)^13,64^. Additionally, *Bacillus* treatments may enhance plant antioxidant activity, gene expression, and synthesis of protective proteins, contributing to stress tolerance overall^72^. It is important to note that no uniformity rates were observed in the performance of the native isolates examined in the experiments. The disparity in their effectiveness as PGPB could be attributed to genetic and environmental factors. According to Samain et al.^73^, the effectiveness of the strains depends on their ability to establish specific interactions with plants and their level of adaptation to adverse conditions.

This is the first study to conduct a comprehensive assessment of the potential of *Bacillus* genus rhizobacteria as promoters of plant growth and mitigators of water stress in chiltepin seedlings *(Capsicum annuum* var. *glabriusculum*). The ability of *Bacillus* isolates to promote chiltepin seed germination and growth was demonstrated both in vitro and in vivo*.* Improvements in germination parameters such as germination rate and speed, as well as vigor and germination index, indicate the potential of these isolates as biostimulants for chiltepin production. Evaluation of drought tolerance revealed that *Bacillus* isolates could mitigate the adverse effects of water stress on chiltepin seedlings. Both under in vitro conditions and in the greenhouse, *Bacillus* treatments improved seedling survival, reduced visual symptoms of stress, and increased relative leaf water content. Together, these findings highlight the potential of *Bacillus* genus rhizobacteria as practical tools for enhancing chiltepin crop production and resilience, providing sustainable alternatives to conventional agricultural inputs, and contributing to food security and environmental sustainability in the study region. However, further studies are needed to fully understand the underlying mechanisms and optimize the application strategies of these biostimulants in field agricultural production systems.

## Methods

### Soil sample collection

Representative soil samples were collected from 15 commercial chili plots from the agricultural zone in Meoquí, Chihuahua, Mexico (28°23´23″ N, 105°37´25″ W) in January 2022. The samples were labeled and transferred to 4 °C for processing.

### Isolation and growth of *Bacillus* spp.

The isolation process of *Bacillus* spp. was carried out in the Applied Microbiology, Phytopathology, and Postharvest Physiology Laboratory (MAFFP) of the School of Agrotechnological Sciences of the Autonomous University of Chihuahua (UACH). The bacteria were isolated according to the methodology described by Astorga et al.^74^. For this, 0.5 g of soil was dissolved in 4.5 mL of 0.85% (w/v) sterile saline solution and kept under constant agitation for 3 min using a vortex. Subsequently, a heat treatment at 80 °C was applied for 10 min in a water bath, followed by serial micro-dilutions in a microplate up to a 10^–6^ dilution factor. Colonies showing the typical macroscopic characteristics of the *Bacillus* genus were subcultured until pure colonies were obtained.

### Morphological and biochemical identification of *Bacillus* spp.

For the identification of the isolates, the hanging drop motility test was conducted according to the methodology of Covadonga & de Silóniz^30^, Gram staining, endospore staining using the Schaeffer-Fulton technique, and potassium hydroxide (KOH) test were performed. In addition, microscopic characteristics and colonial morphology were reviewed as described by Garrity et al.^75,76^

### Molecular identification of *Bacillus* spp.

A polymerase chain reaction (PCR) was carried out to amplify the 16S rRNA fragment for molecular identification. Total genomic DNA extraction from the rhizobacteria was performed using the modified phenol–chloroform method, according to Bardakci & Skibinski^76^. The concentration and purity of DNA from the samples were determined with a biospectrophotometer using the SW Version: 4.2.3.0 processor. The reaction mixture was prepared with the following components from Thermo Scientific (Massachusetts, USA): 1 × Taq buffer with KCl, 2.5 mM MgCl2, 0.2 mM dNTP'S, 0.25 U Taq DNA polymerase, and H2O. Subsequently, 2.5 μL of primers 27F- 5' AGA GTT TGA TCC TGG CTC AG 3' and 1492R- 5' TAC GGT TAC CTT GTT ACG ACT T 3' (Integrated DNA Technologies, Iowa, USA) and DNA at a concentration of 10 ng/μL were added. The PCR was performed with the following amplification program: an initial denaturation for 10 min at 95 °C followed by 30 amplification cycles (1 min of denaturation at 94 °C, 1 min of annealing at 50 °C, and 1.5 min of extension at 72 °C) followed by a final 10-min extension at 72 °C^77^, The amplified fragments were sent to Macrogen Crop (Rockville, Maryland, USA) for sequencing using the Sanger method. The sequences were submitted to the NCBI (National Center for Biotechnology Information) to obtain the accession number. Finally, a search for homologous sequences was conducted using the nBLAST tool from the gene bank.

### Seed inoculation

Seeds of *C. annuum* var*. glabriusculum* were used, extracted from wild plants from the locality of Chínipas, Chihuahua (27 24′ 0 N, 108 32′ 0 W). They were selected and disinfected in a 5% (v/v) NaClO solution under constant agitation for 5 min, followed by 3 rinses with distilled water (H_2_Od). They were then kept in sterile H_2_Od for 24 h and treated with bacterial solutions (1 × 10^8^ CFU/mL) under constant agitation at 150 rpm / 28 °C for 1 h, after which the bacterial solution was discarded. The plates were placed in a germination chamber with a photoperiod of 16 h of light at 28 °C and 8 h of darkness at 25 °C.

### Germination parameters

To determine the effect of the isolates on germination promotion, 25 chiltepin seeds were inoculated with 12 *Bacillus* spp. isolates. The commercial isolate QST 713 of *B. subtilis* (Serenade® ASO = BsQ) was used as a positive control, and seeds without treatment were used as a negative control (*n* = 4). Germination was monitored 19 days after sowing (DAS) to determine germination parameters and indices (Table 8). In addition, vegetative parameters (fresh weight, stem length, root, stem diameter) were evaluated in 5 seedlings.

**Table 8:** Germination parameters and indices.

Germination Parameter	Key	Formula		References
Germination rate	GR (%)	(N/A) * 100	N is the seed's germinated number, and A is the seed's total number	Labouriau,^78^
Mean germination time	MGT (days)	n_t_ * t_i/total_	n_t_ is the seeds germinated number in a time interval, it is the time interval y n _total_ is the seeds germinated number	
Mean speed of germination	MSG (semillas/dia)	1/t	G_1_, G_2_, G_n_ is the seedling's number calculated in the first, second, and last count, and N_1_, N_2_, N_n_ is the day's number from sowing to the first, second, and final count	
Germination speed index	GSI	(G_1_/N_1_) + (G_2_/N_2_) + …. + (G_n_/N_n_)		
Vigor index	VI	GR × height (mm)	Abdul & Anderson^79^	
Slenderness index		Height (cm) / Diameter_stem_ (mm)	Sáenz et al*.* ^42^	
Germination index	GI	(GR × Root length / GR _Control_ × Root length _Control_) × 100	Zucconi et al*.* ^40^	

### Beneficial plant growth promoting traits

The solubilization of phosphorus, ammonia production, siderophore production, zinc solubilization, and exopolysaccharide production were evaluated in the 12 isolated *Bacillus* spp.

#### Phosphorus solubilization

The phosphorus solubilization capacity of the *Bacillus* spp. isolates were assessed by inoculating 5 μL of growth (1 × 10^8^ CFU/mL) on Pikovskaya medium (*n* = 5). The solubilization index (SI) was determined according to the following formula^80^.

$${\text{SI}} = {\text{colony diameter}}\left( {{\text{cm}}} \right) + {\text{halo zone diameter}}\left( {{\text{cm}}} \right)/{\text{colony diameter}}\left( {{\text{cm}}} \right).$$

#### Ammonium production

Ammonia production was evaluated using a colorimetric method through the Nessler assay^81^adapted to a microplate adjusting to a total volume of 200 μL (*n* = 4).

#### Siderophores production

Siderophore production was assessed using the Chrome Azurol Sulfonate (CAS) technique developed by Schwyn & Neilands^82^ through pinprick inoculation (*n* = 5).

#### Zinc solubilization

The zinc solubilization capacity was evaluated on Bunt & Rovira^83^ plate culture medium supplemented with 0.1% ZnO as an insoluble zinc source (*n* = 5).

#### Exopolysaccharides production

Exopolysaccharide production was determined by the gravimetric method (*n* = 4)^84^.

### *Drought tolerance under *in vitro* conditions*

#### Drought tolerance potential of *Bacillus* isolates

To assess the resistance of *Bacillus* spp. isolates to different concentrations of polyethylene glycol, the technique described by Yadav et al*.*^85^ was used. For this, a microplate with 180 μL of nutrient broth culture medium supplemented with polyethylene glycol at concentrations of 10, 20, 30, 40, and 50% was used, along with control without PEG (P10, P20, P30, P40, P50, and P0, respectively). 5 mL of each bacterial growth (1 × 10^8^ CFU/mL) was centrifuged at 5000 rpm, 4 °C for 5 min. The supernatant was then decanted, and the pellet was resuspended in 0.5 mL of 0.85% sterile saline solution. Next, the wells were inoculated with 20 μL of the bacterial suspension and kept in an incubator at 28 °C for 96 h (*n* = 3). To assess bacterial growth, an OD measurement at 600 nm was taken, and the survival rate of each bacterium was calculated relative to the control (P0). To qualitatively evaluate viability, 10 μL of resazurin (0.03 M) was applied to each well; a color change from blue to pink shades indicated cell viability^86^.

#### In vitro* evaluation of stress induction in chiltepin seedlings*

Seedlings 23 days after sowing (DAS) inoculated with the bacterial solutions (1 × 10^8^ CFU/mL) were transferred to tubes containing 1 mL of PEG (30%) (*n* = 9). After 5 days, the survival rate of seedlings and vegetative parameters such as height, root length, stem diameter, and slenderness index were evaluated as described in the previous sections.

### Growth promotion and drought tolerance in greenhouse conditions

In the growth promotion and drought tolerance assays under greenhouse conditions, the strains Bt24-4, Bc25-7, and Bc30-2 were used. These strains were selected due to their outstanding performance in the germination and in vitro water stress assays. During the germination of chiltepín, they showed the best results in GR, the germination index, and GSI. Additionally, these strains were grouped within the two highest-performing groups in analyzing vegetative parameters using PCA-cluster analysis. Moreover, they also induced a high survival rate and a low slenderness index in chiltepín seedlings in the in vitro water stress experiment.

#### Growth promotion

For this assay, seedlings were produced from disinfected seeds, and transplanting took place 60 days after sowing (DAS) with 3–4 true leaves. The seedlings were placed in a greenhouse, and temperature and humidity were monitored throughout the experiment. Irrigation was carried out every 48–72 h with distilled water (H_2_Od,) and a Steiner nutrient solution (pH 6.0; EC 1250 µS) was applied^87^. The first inoculation was done on the day of transplant by applying 5 mL of a bacterial suspension (1 × 10^8^ CFU/mL) to the base of each seedling, and this was repeated three times every 15 days. Serenade® was used as a positive control, and seedlings treated with water were used as a negative control (*n* = 6). The evaluation of vegetative parameters was carried out 37 days after transplant (DAT), considering height, leaf count, leaf area^88^, stem diameter, root length, fresh and dry biomass, pigment content (chlorophyll a, b, and carotenoids)^89^

#### Drought tolerance in greenhouse conditions

This experiment used the commercial product Xp Amino® and the Serenade® isolate as positive controls. Otherwise, seedlings without treatment were used as a negative control, and seedlings without treatment and stress were used as an absolute control (*n* = 6). 75 DAS seedlings inoculated with *Bacillus* spp. isolates were subjected to water stress by withholding water for seven days^72^. To do this, the seedlings were watered to saturation, submerging the vessels for 30 s in 500 mL of tap water. Excess water was then decanted, and after 1 h, the substrate's moisture was determined (3 Way Soil Meter). The leaves proline content and relative water content (RWC) were determined for the evaluation. The total proline estimation was carried out using the method of Bates et al.^90^; 0.5 g of leaf tissue was homogenized in 10 mL of 3% aqueous sulfosalicylic acid and passed through Whatman Filter Paper 1. Next, 2 mL of the filtrate was taken, and 2 mL of concentrated glacial acetic acid and 2 mL of acid ninhydrin (1.25 g ninhydrin, 30 mL glacial acetic acid, and 20 mL 6 M phosphoric acid) were added. The mixture was placed in a water bath at 100 °C for 1 h, and the reaction was stopped on ice. Subsequently, 4 mL of toluene was added, and the absorbance was read at 520 nm. The total proline content estimation (µM/g of fresh plant material) was made using a standard L-proline curve. The relative water content (RWC) in the leaves was determined using the following formula^91^:

$${\text{RWC}}\left( \% \right) = \left[ {\left( {{\text{FW}}{-}{\text{DW}}} \right)/\left( {{\text{WT}}{-}{\text{DW}}} \right)} \right] \times 100$$

where: FW is the fresh weight after seedling harvesting, WT is the weight of leaves saturated when submerged in H_2_O for 4 h (turgid weight), and DW is the constant dry weight.

### Statistical analysis

A completely randomized design was established with a single factor (*Bacillus* spp.). To select the isolates with the highest growth promotion potential, a principal component analysis was carried out with vegetative parameters and the grouping of the isolates using k-means (3 clusters). The data were analyzed with an ANOVA and mean separation using the Scott Knott test (*α* = 0.05). The analyses were performed using the statistical software InfoStat 2020d and Minitab 20.3.

### Supplementary Information

Supplementary Information 1.

Supplementary Information 2.

## Data Availability

All data generated or analysed during this study are included in this published article and [Raw Data availability.exe]. The authors declare that the wild plant collection used in this investigation was not considered a plant population risk. Wild plants used in this manuscript are not considered threatened plants according to Official Mexican Standard NOM-059-SEMARNAT-2010, Environmental Protection—Native species of Mexico of flora and fauna—Risk categories and specifications for their inclusion, exclusion or change—List of species at risk (last update 2022).
