# Supplementary material for: Enhancing drought stress tolerance and growth promotion in chiltepin pepper (Capsicum annuum var. glabriusculum) through native Bacillus spp
Source: Sci Rep. 2024 Jul 4;14:15383. doi: 10.1038/s41598-024-65720-y (PMC11224271; doi:10.1038/s41598-024-65720-y)
Supplement: Supplementary file 2 — Supplementary Information 2. [file 41598_2024_65720_MOESM2_ESM.docx]

**Supplementary Information**


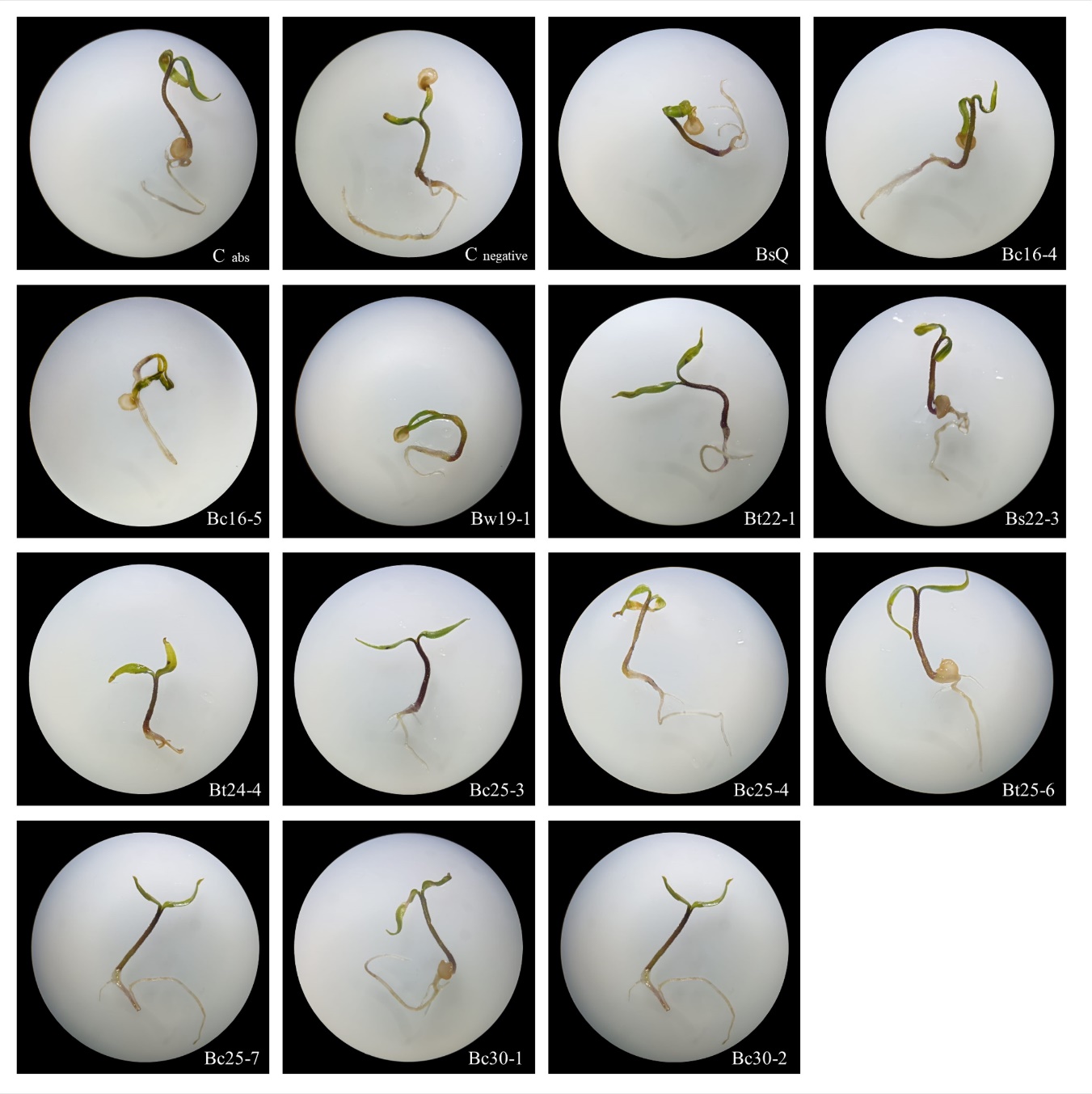


**Supplementary Figure S1.** *C. annum* var. *glabriusculum* seedlings treated with *Bacillus* spp. on day 7 after induction of water stress with polyethylene glycol (30%) *in vitro.* C_abs_ = absolute control, negative control, BsQ = *B. subtilis* QST 713 ®, Bc16-4-Bc30-2 = *B. cereus* isolates, Bt22-1-Bt25-6 = *B. thuringiensis* isolates, Bs22-3 = *B. subtilis* isolate, Bw19-1 = *B. wiedmannii* isolate.

*
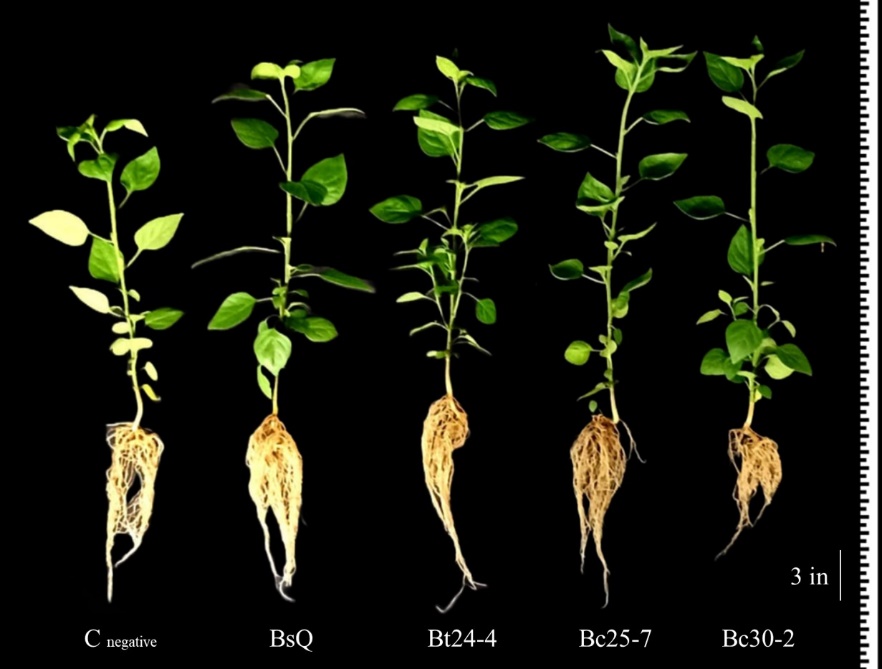
*

**Supplementary Figure S2.** Growth promotion in *C. annuum* var. *glabriusculum* seedlings treated with *Bacillus* spp. and developed in greenhouse conditions on day 42 after transplant. Negative control, BsQ = *B. subtilis* QST 713®, Bc25-7-Bc30-2 = *B. cereus* isolates, Bt24-4 = *B. thuringiensis* isolate.


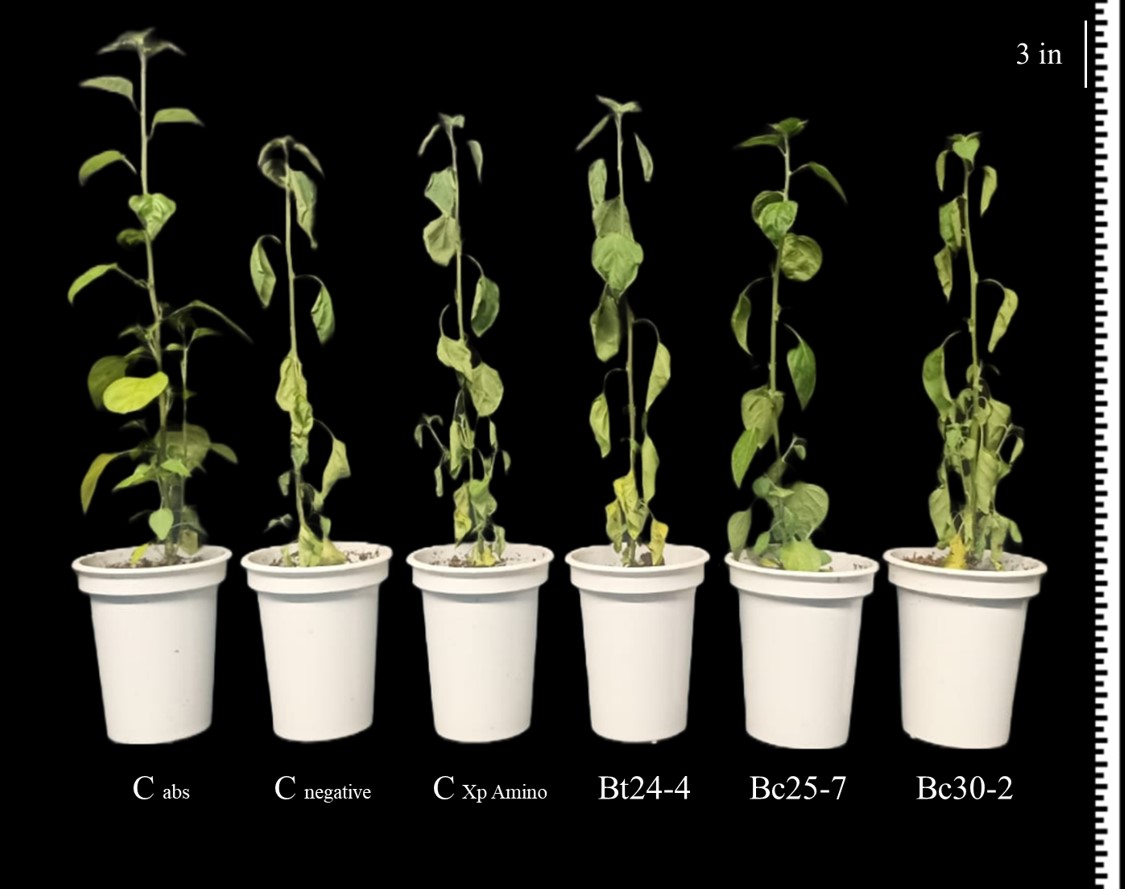


**Supplementary Figure S3.** *C. anuum* var. *glabriusculum* seedlings treated with *Bacillus* spp.and developed in greenhouse conditions on day 7 after withholding water.*.* C_abs_ = absolute control, negative control, C_Xp Amino_ = Xp Amino® (commercial product to improve stress resistance), Bc25-7-Bc30-2 = *B. cereus* isolates, Bt24-4 = *B. thuringiensis* isolate.
